# The Potential of Five Immune-Related Prognostic Genes to Predict Survival and Response to Immune Checkpoint Inhibitors for Soft Tissue Sarcomas Based on Multi-Omic Study

**DOI:** 10.3389/fonc.2020.01317

**Published:** 2020-07-24

**Authors:** Hui-Yun Gu, Lu-Lu Lin, Chao Zhang, Min Yang, Hou-Cheng Zhong, Ren-Xiong Wei

**Affiliations:** ^1^Department of Spine and Orthopedic Oncology, Zhongnan Hospital of Wuhan University, Wuhan, China; ^2^Department of Pathology and Pathophysiology, School of Basic Medicine, Wuhan University, Wuhan, China; ^3^Center for Evidence-Based Medicine and Clinical Research, Taihe Hospital, Hubei University of Medicine, Shiyan, China; ^4^Department of Oncology, Taihe Hospital, Hubei University of Medicine, Shiyan, China

**Keywords:** immune-related prognostic genes (model), tumor immune microenvironment, somatic variants, DNA methylation, multi-omic study

## Abstract

Low response rates to immunotherapy have been reported in soft tissue sarcoma (STS). There are few predictive biomarkers of response, and the tumor immune microenvironment associated with progression and prognosis remains unclear in STS. Gene expression data from the Cancer Genome Atlas were used to identify the immune-related prognostic genes (IRPGs) and construct the immune gene-related prognostic model (IGRPM). The tumor immune microenvironment was characterized to reveal differences between patients with different prognoses. Furthermore, somatic mutation data and DNA methylation data were analyzed to understand the underlying mechanism leading to different prognoses. The IGRPM was constructed using five IRPGs (IFIH1, CTSG, STC2, SECTM1, and BIRC5). Two groups (high- and low-risk patients) were identified based on the risk score. Low-risk patients with higher overall survival time had higher immune scores, more immune cell infiltration (e.g., CD8 T cell and activated natural killer cells), higher expression of immune-stimulating molecules, higher stimulating cytokines and corresponding receptors, higher innate immunity molecules, and stronger antigen-presenting capacity. However, inhibition of immunity was observed in low-risk patients owing to the higher expression of immune checkpoint molecules and inhibiting cytokines. High-risk patients had high tumor mutation burden, which did not significantly influence survival. Gene set enrichment analysis further revealed that pathways of cell cycle and cancers were activated in high-risk patients. DNA methylation analysis indicated that relative high methylation was associated with better overall survival. Finally, the age, mitotic counts, and risk scores were independent prognostic factors for STS. Five IRPGs performed well in risk stratification of patients and are candidate biomarkers for predicting response to immunotherapy. Differences observed through the multi-omic study of patients with different prognoses may reveal the underlying mechanism of the development and progression of STS, and thereby improve treatment.

## Introduction

Unlike cancers with epithelial origins, soft tissue sarcoma (STS) evolved from mesenchymal tissues in different anatomical sites (1). Despite its lower incidence vs. cancers, STS with high aggressive behavior was responsible for 5,270 deaths according to the 2019 Cancer Statistics (2). Occurrence of STS in the limbs increases the risk of disability in patients (3). Moreover, STS is characterized by high rates of relapse (4). Therefore, the treatment of STS is a challenge to most clinicians.

Patients with early-stage and localized STS (5, 6) can recover from radical surgical resection and achieve higher survival rates. However, patients with metastatic and recurrent STS are linked to rapid progression of disease and death due to poor response to surgical techniques and adjuvant radiotherapy (7). Conventional treatment does not meet the requirements for longer survival time and higher quality of life. An increasing number of studies revealed that the tumor microenvironment and the expression of immune checkpoint molecules accelerated the progression of cancers (8–10). The use of immune checkpoint inhibitors markedly improved the prognosis of cancers (e.g., melanoma) (11, 12). Based on the immune-related pathogenesis in cancers, the use of immunotherapy may promote survival in STS. Recent clinical cases reported favorable response to immune checkpoint inhibitors in classic Kaposi sarcoma (13) and myxoid chondrosarcoma (14). Nevertheless, there is insufficient evidence regarding the efficacy of immunotherapy in STS. Therefore, studies investigating the immune microenvironment or immune gene-related prognostic biomarkers, which have been identified in cervical cancer (15), lung adenocarcinoma (16), and cancers of the digestive system (17) are warranted. Such studies will assist in understanding the effect of immune infiltration on STS and predict response to immunotherapy, thereby improving efficacy against STS.

The aim of the present study was to identify immune gene-related prognostic biomarkers and construct a prognostic model to determine patients with better response to immunotherapy for precision treatment in STS. Moreover, integrated analysis of multi-omic data in patients with different prognoses may elucidate the mechanism involved in tumorigenesis, metastasis, and high aggressive behavior of STS.

## Materials and Methods

### Collection and Preprocessing of Gene Expression and Clinical Data

The latest version of normalized gene expression data (07-20-2019) in the Cancer Genome Atlas (TCGA) database were downloaded from the UCSC (University of California, Santa Cruz) Xena browser (https://gdc.xenahubs.net). Raw gene expression data (GSE21050) (18) were also downloaded from the Gene Expression Omnibus (GEO) database. Subsequently, the gene expression profiles were preprocessed. The “RMA” algorithm (19) was used to process the GSE21050. During the procedure of probe mapping to gene symbols, mean values were maintained when multiple probes shared the same gene symbol. The gene symbols with mean expression value in all samples <0.5 were removed (20). For the subsequent analyses, we selected common genes with top 25% variances in TCGA and GEO datasets (21). In addition, clinical data of the TCGA and GEO samples were downloaded and preprocessed.

### Weighted Gene Co-expression Network Analysis (WGCNA)

WGCNA, a new bioinformatics method, is effective in processing gene expression, proteomic, and metabolomic datasets (22, 23). It has been applied to the identification of potential crucial biomarkers in many types of diseases (24, 25) and key genes associated with phenotypic traits (26). There was no information regarding the survival status in the GSE21050 dataset. Therefore, WGCNA was performed to identify prognostic genes based on the expression matrix obtained from TCGA database. The gene expression matrix of genes with top 25% variance was used to construct a gene co-expression network and identify modules. Subsequently, we related modules to clinical information for the detection of modules highly associated with survival. All these procedures were performed using the “WGCNA” package (22) in R 3.5.3 software. Function enrichment analyses were conducted using the “clusterProfiler” package (27) in R software to further determine whether interesting modules were associated with survival. Following the completion of WGCNA, genes identified in the survival-related modules (interesting modules) were extracted as the preliminary immune-related prognostic genes (IRPGs) in STS.

### Identification and Validation of the Immune Gene-Related Prognostic Model (IGRPM)

Immune-related genes (IRGs) identified in the Immunology Database and Analysis Portal (ImmPort) database (28) were overlapped with the prognostic genes detected in the WGCNA. The expression matrix containing common immune genes from TCGA and GEO datasets was processed through the “sva” package of R software (29) to remove the batch effect. Subsequently, it was used to identify and validate IRPGs and construct the prognostic model. Firstly, univariate Cox regression analysis of common immune genes was performed based on the “survival” package of the R software. Least absolute shrinkage and selection operator (LASSO) regression analysis (30) was applied for genes with *p* < 0.05 in the univariate Cox regression analysis. Through the 1000 cross-validations method, more reliable IRPGs would be obtained using the “glmnet” and “survival” packages (31). Genes identified from the LASSO regression analysis were further determined via multivariate Cox regression analysis. The genes that demonstrated significance in the multivariate Cox regression analysis were considered the IRPGs in this study. According to the coefficient of IRPGs in the multivariate Cox regression analysis, the “predict” function in the “survMisc” package was used to construct the IGRPM and compute the risk score for each patient. Based on the median risk scores, patients with risk scores more than the median risk scores were classified into high-risk groups. Similarly, patients with risk scores less than the median were classified into low-risk groups. Subsequently, we plotted the receiver operating characteristic (ROC) curve using the “survivalROC” package and performed overall survival (OS) analysis to evaluate the IGRPM. Differences in gene expression, survival status, and risk scores between the high- and low-risk groups were also visualized to evaluate the prognostic model. Finally, the GSE21050 dataset was used to validate the accuracy of the model based on the same cutoff value applied to the TCGA dataset. Owing to the lack of survival status in this dataset, the rates of metastasis-free survival were used as OS rates to validate the IGRPM.

### Immune Infiltration Analysis

The immune microenvironment was investigated in this study. An algorithm using expression data for the estimation of stromal and immune cells in malignant tumors (ESTIMATE) was applied (32). The stromal score and immune score for each patient with STS were computed based on specific gene expression signatures of stromal and immune cells, and single-sample gene set enrichment analysis via the “estimate” package in the TCGA and GSE21050 datasets (32). Differences in stromal and immune cell infiltration between high- and low-risk patients were visualized via the “ggpubr” package (https://CRAN.R-project.org/package=ggpubr). Furthermore, the correlation of risk scores and immune scores was explored to reveal the effect of immune infiltration on prognosis. Subsequently, the CIBERSORT algorithm based on 100 permutations was used to estimate the proportions of 22 types of immune cells following the official manual provided in the CIBERSORT website (http://cibersort.stanford.edu/) (33). In addition, based on previous studies, immune-related molecules and other immune microenvironment components except immune cells (i.e., chemokines, interleukins, interferons, other cytokines, corresponding receptors of the aforementioned molecules, innate immunity molecules, immune inhibitors including common immune checkpoints, immune stimulators, and antigen-presenting molecules) were further analyzed to understand immune infiltration in STS (34–36). A *p* < 0.05 denoted statistically significant difference between high- and low-risk patients.

### Analysis of Somatic Variants

Notably, gene mutations may lead to neoantigen epitopes and influence the components of immune microenvironments (16). Mutation data from the VarScan2 Variant Aggregation and Masking were downloaded through the UCSC Xena website. The “maftools” package with functions for summarizing, analyzing, and visualizing mutation data was used to analyze somatic variants (37). Firstly, the overall mutation status, as well as the corresponding risk and immune scores were determined in all patients. Differential mutations were investigated in patients with different risk and immune scores to identify the crucial gene mutations associated with prognosis and immune filtration, and demonstrate the relationship between immune filtration and prognosis in STS. Finally, the tumor mutation burden (TMB) was calculated between high- and low-risk patients based on a previous study (38).

### Gene Set Enrichment Analysis (GSEA)

We performed the GSEA to elucidate the underlying mechanism involved in immune infiltration and high aggressive behavior in STS. The Kyoto Encyclopedia of Genes and Genomes (KEGG) pathway was obtained via the GSEA 4.0.1 software (39). Based on the ranking of normalized enrichment scores, the top 10 terms were displayed to identify differences in biological pathways and behaviors between high- and low-risk patients.

### DNA Methylation Analysis

Illumina Human Methylation 450 k data were download from the UCSC Xena website. Using the “ChAMP” package, we filtered the non-cg probes; the CpG falls near a single-nucleotide polymorphism; the probe aligns to multiple locations; and from the X and Y chromosomes (40). Subsequently, the remaining probes were used to conduct the differential analysis between high- and low-risk patients. The mean methylation value of the differential probes was utilized to display the methylation of the promoter, body, 3′untranslated regions (3′UTR), and intergenic regions (IGR) in high- and low-risk patients. Furthermore, the association between methylation and prognosis was investigated. Finally, the differential probes were enriched using the “missMethyl” package to observe potential mechanisms involved in different prognoses.

### Identification of Independent Prognostic Factors (IPF) for STS

Clinical factors (i.e., age, sex, margin status, and metastatic diagnosis), mitotic counts, total necrosis percent of tumors, risk scores, and immune scores were included in the univariate and multivariate Cox regression analyses to further evaluate the prognostic model and identify IPF. Only factors with *p* < 0.05 in both analyses were considered IPF. In addition, the prognostic ability of risk scores and immune scores was evaluated via ROC curves.

## Results

### Collection and Preprocessing of Gene Expression and Clinical Data

The datasets of TCGA contained 263 samples with STS and two matched controls. After removing the two matched controls, the gene expression data were analyzed. The GSE21050 dataset comprised 310 samples with STS. Using a preliminary filter, 4,113 and 5,046 genes with top 25% variances were obtained from TCGA and GEO datasets, respectively. Notably, they shared 2,447 genes with the WGCNA. The corresponding clinical data of patients were matched to their corresponding gene expression profiles for the subsequent analysis. A total of 256 patients with survival information were included in the survival or prognosis analysis in TCGA dataset. The patient (GSM525864) lacking information regarding survival and metastasis were not included in these analyses of the GSE21050 dataset.

### Two Modules Containing 1,141 Genes Were Associated With Survival

**Figure 1:**
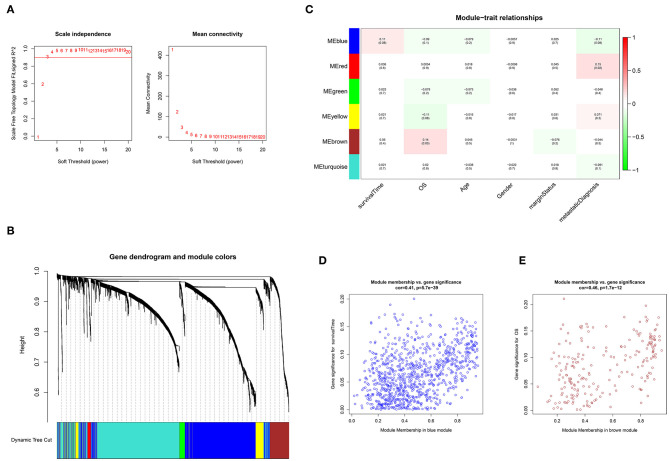
Weighted gene co-expression network analysis (WGCNA). **(A)** Selection of the optimal soft threshold power, β (optimal β = 3, scale free topology index, *R*^2^ = 0·9). **(B)** Module identification using the Dynamic Tree Cut method. The different color bands provide a simple visual comparison of module assignments. **(C)** Module–trait relationship. Six modules were identified and related to clinical traits. Each cell represented the correlation (and *p*-value) of the module with the corresponding clinical trait. OS represented the overall survival status (alive or dead). **(D)** Identification of genes with high significance and module membership in the survival time-related blue module. **(E)** Identification of genes with high significance and module membership in the OS-related brown module.

As shown in Figure 1A, an adjacency matrix was constructed based on the soft threshold power β (optimal β = 3; *R*^2^ = 0·9), determined through the scale-free topology criterion. Figure 1B illustrates the identified modules obtained using the Dynamic Tree Cut method. Subsequently, the identified modules were related to the clinical data. Compared with other modules, two modules were highly associated with survival (Figure 1C). The blue module significantly influenced the survival time of patients with STS (Pearson's correlation between blue module and the trait of survival time: 0.11, *p* = 0.08). A similar relationship was observed in the brown module associated with the OS status (Pearson's correlation between brown module and the trait of OS status: 0.14, *p* = 0.03). Furthermore, genes in the blue and brown modules exhibited high positive correlations with survival (Pearson's correlation between gene significance and module membership: 0.41, *p* = 5.7e−39; Pearson's correlation between gene significance and module membership: 0.46, *p* = 1.7e−12, respectively) (Figures 1D,E). Function annotation revealed that 929 and 212 genes in the blue module and brown module were mainly involved in the immune process and cell cycle process, respectively. These findings indicated that genes in these two modules highly influenced survival in STS (*p* < 0.05) (Figure S1).

### Identification and Validation of IGRPM

**Figure 2:**
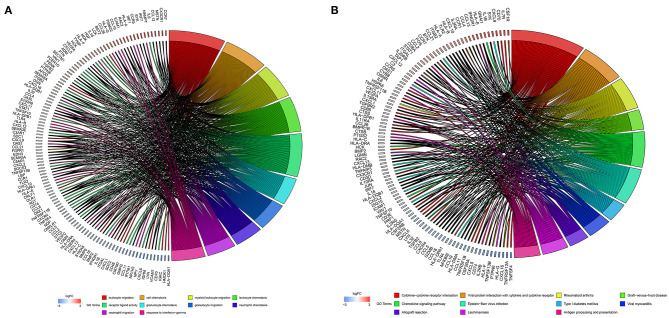
Function enrichment analyses for the overlapped genes from the WGCNA and the Immunology Database and Analysis Portal (ImmPort) database. **(A)** Gene Ontology (GO) analysis for the immune genes. **(B)** The Kyoto Encyclopedia of Genes and Genomes (KEGG) pathways for immune genes.

**Figure 3:**
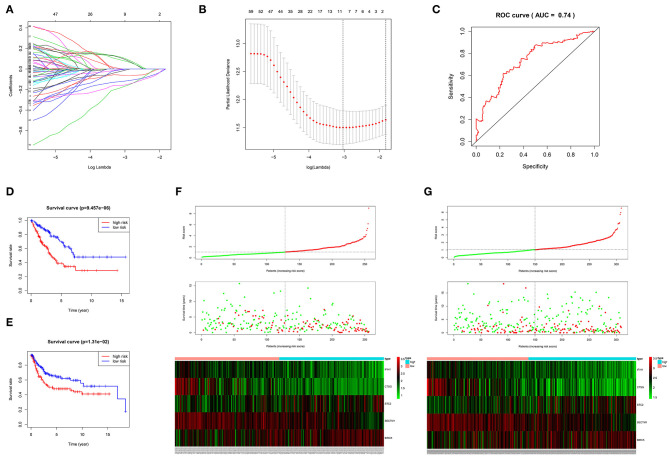
Construction, validation, and assessment of the prognostic model. **(A,B)** Identification of prognostic genes using the least absolute shrinkage and selection operator (LASSO) regression analysis. **(A)** Coefficients of the LASSO regression analysis. **(B)** Selection of tuning parameters based on the 1000 cross-validations method. **(C)** The receiver operating characteristic (ROC) curves of the prognostic model in TCGA dataset. The survival curve for high- and low-risk patients in TCGA **(D)** and GSE21050 **(E)** datasets. Differences in risk score, survival time, and gene expression of the optimal immune-related prognostic genes (IRPGs) between high- and low-risk patients from TCGA **(F)** and GSE21050 **(G)** datasets.

The 1,141 genes obtained from the WGCNA and the IRGs from the ImmPort database shared 207 IRGs, which were involved in immune activity via multiple pathways (*p* < 0.05) (Figure 2). These 207 IRGs were subsequently analyzed to identify optimal IRPGs. Firstly, 81 IRGs associated with survival from univariate Cox regression analysis were obtained based on *p* < 0.05. LASSO regression analysis displayed that nine IRGs (i.e., CD1C, C-X-C motif chemokine ligand 2 [CXCL2], interferon induced with helicase C domain 1 [IFIH1], cathepsin G [CTSG], stanniocalcin 2 [STC2], secreted and transmembrane 1 [SECTM1], baculoviral IAP repeat containing 5 [BIRC5], endothelin 3 [EDN3], and nuclear receptor subfamily 1 group H member 3 [NR1H3]) were associated with survival based on the 1000 cross-validations approach (Figures 3A,B). Five optimal IRPGs (i.e., IFIH1, CTSG, STC2, SECTM1, and BIRC5) were obtained from the multivariate Cox regression analysis (Table 1). Subsequently, we used the “predict” function to construct the prognostic model with an area under the curve of 0.74 in 5-year survival rates (Figure 3C). High- and low-risk patients, determined according to their risk scores, had significant differences in survival rates in TCGA (*p* = 9.457e−06) (Figure 3D) and GSE21050 (*p* = 1.31e−02) datasets (Figure 3E). The prognostic model was further assessed and validated (Figures 3F,G).

**Table 1:** The multivariate Cox regression analysis of genes for overall survival.

**Genes**	**Overall survival**				
	**coef**	**HR**	**95% CI**		***p*-value**
IFIH1	−0.373	0.689	0.505	0.939	0.018
CTSG	−0.215	0.806	0.671	0.968	0.021
STC2	0.195	1.215	1.027	1.438	0.023
SECTM1	−0.259	0.772	0.631	0.944	0.012
BIRC5	0.229	1.258	1.033	1.530	0.022

*coef, coefficient; HR, hazard ratio; CI, confidence interval*.

### Relatively Higher Immune Activation Was Observed in Low-Risk Patients

**Figure 4:**
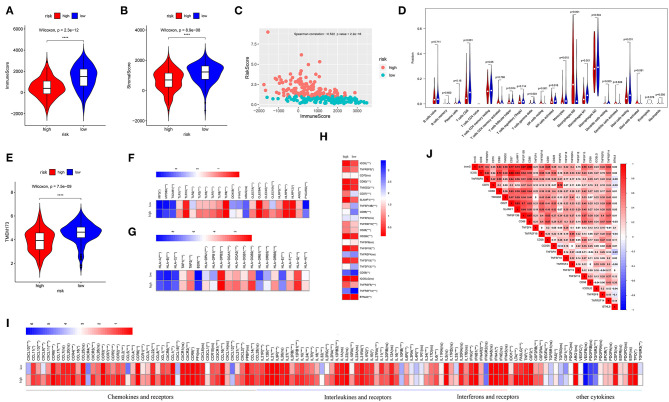
Analysis of immune infiltration in the TCGA dataset. Comparison of the immune scores **(A)** and stromal scores **(B)** between high- and low-risk patients. **(C)** Spearman's correlation of risk scores with the immune scores. **(D)** Comparison of 22 types of immune cells in high- and low-risk patients. **(E)** Comparison of the TMEM173, the initiation molecule of innate immunity between high- and low-risk patients. Comparison of the other molecules of innate immunity **(F)**, major histocompatibility complex (MHC) molecules **(G)**, and immune-stimulating molecules **(H)**, and cytokines **(I)** between high- and low-risk patients. Low-risk patients exhibited high expression of most of these molecules. Relationships of immune infiltration (immune scores) and immune-stimulating molecules **(J)**. Immune infiltration was positively correlated with most immune-stimulating molecules. ImmS represents the immune scores. ****p < 0.0001; ***p < 0.001; **0.001 < *p* < 0.01; *0.01 < *p* < 0.05; ns (not significant), *p* > 0.05.

Estimated immune and stromal scores, immune cells, and immune-related molecules were assessed for the level of immune infiltration between high- and low-risk patients. Higher immune scores and stromal scores were observed in low-risk patients in TCGA (*p* = 2.3e−12; *p* = 8.9e−08, respectively) (Figures 4A,B) and GSE21050 (*p* = 2.9e−16; *p* = 6.4e−15, respectively) (Figures S2A,B) datasets. Strong negative correlations between the immune scores and risk scores indicated that higher immune filtration was a potential protective factor for survival (Spearman's correlation: −0.522, *p* < 2.2e−16; Figure 4C and Spearman's correlation: −0.540, *p* < 2.2e−16; Figure S2C). By analyzing the proportion of immune cells in TCGA dataset, a greater number of immune cells (i.e., CD8 T cells, gamma delta T cells, activated natural killer (NK) cells, monocytes, M1 macrophages, and resting mast cells) were found in low-risk patients (*p* < 0.05, Figure 4D). Similarly, more CD8 T cells, follicular helper T cells, gamma delta T cells, activated NK cells, M1 macrophages, and resting mast cells were observed in low-risk patients in the GES21050 dataset (*p* < 0.05, Figure S2D). In addition, immune-related molecules demonstrated that low-risk patients had higher immune activation. High expression of innate immune modules, especially TMEM173(STING1) (Wilcoxon rank-sum test: *p* = 7.5e−09, Figure 4E and *p* = 4.8e−09, Figure S2E), was noted in low-risk patients (Figure 4F, Figure S2F). Moreover, low-risk patients exhibited stronger antigen-presenting capacity, mainly measured through the higher expression of major histocompatibility complex (MHC) molecules (e.g., MHC I, MHC II, etc.) (Figure 4G, Figure S2G). A similar tendency was observed in comparisons of immune-stimulating molecules, cytokines, and corresponding receptors between patients with different risks (Figures 4H,I, Figures S2H,I). Finally, positive correlations between immune scores and immune-stimulating molecules further supported and explained the above findings (Figure 4J, Figure S2J).

### Low-Risk Patients Showed Immunosuppression

**Figure 5:**
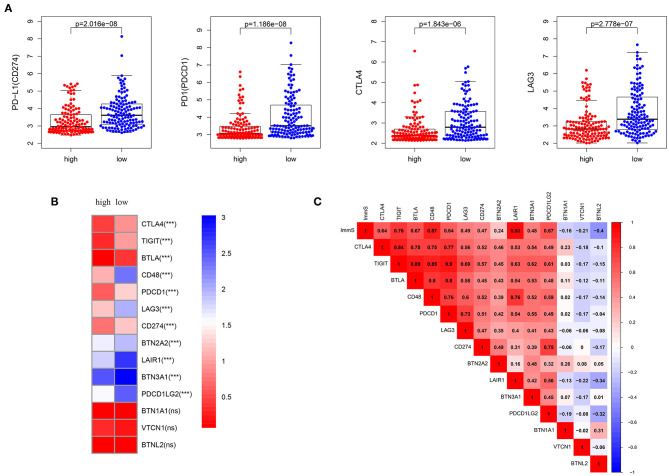
Inhibition of immunity in low-risk patients from TCGA dataset. **(A)** Differences in common immune checkpoint molecules between high- and low-risk patients. **(B)** Differences in immune-inhibiting molecules between high- and low-risk patients. **(C)** Relationships of immune infiltration (immune scores) and immune-inhibiting molecules. Immune infiltration was positively correlated with most immune-inhibiting molecules. ImmS represents the immune scores. ***p < 0.001; ns (not significant), *p* > 0.05.

Characterized by higher immune activation, low-risk patients from TCGA also showed immunosupression. Immunosuppression in low-risk patients was verified through immune-inhibiting molecules, especially common immune checkpoints, such as PD-L1 (CD274), PD1 (programmed cell death 1 [PDCD1]), cytotoxic T-lymphocyte associated protein 4 [CTLA4], and lymphocyte activating 3 [LAG3] (Figures 5A,B, Figures S3A,B). There were no differences in the expression level of these four immune checkpoints between the sexes (Figure S4). In addition, higher levels of inhibiting cytokines (e.g., interleukin 10) were present in the tumor microenvironment of low-risk patients (Figure 4I, Figure S2I). The strong correlations noted between immune scores and most immune-inhibiting molecules revealed that low-risk patients exhibited higher immune infiltration and immunosuppression, which increased the risk of immune escape of STS (Figure 5C, Figure S3C).

### Analysis of Somatic Variants

**Figure 6:**
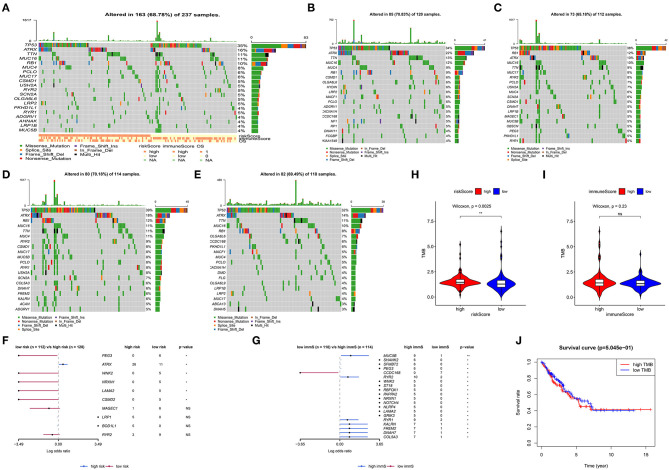
Analysis of somatic variants in soft tissue sarcoma (STS). Top 20 gene mutations in all patients **(A)**. Top 20 gene mutations in high- **(B)** and low-risk patients **(C)**. Top 20 gene mutations in patients with high **(D)** and low **(E)** immune scores. Comparison of high- with low-risk patients **(F)**, as well as patients with high and low immune scores **(G)**. **(H)** Comparison of the log(TMB+1) in high- and low-risk patients. **(I)** Comparison of the log(TMB+1) in patients with high and low immune scores. **(J)** Survival curve for patients with high and low TMB. TMB, tumor mutation burden. **0.001 < *p* < 0.01; *0.01 < *p* < 0.05; ns (not significant), *p* > 0.05.

Gene mutations in STS were also studied. As shown in Figure 6A, 68.78% of the top 20 mutations occurred in all patients, with missense mutation being the most common type. Of note, different types of mutations were observed between high- and low-risk patients. Tumor protein p53 (TP53), ATRX, titin (TTN), mucin 16 (MUC16), and RB transcriptional corepressor 1 (RB1) were the most commonly mutated genes (>10% mutation rate). Obvious differences in the top 20 mutated genes between high- and low-risk patients are illustrated in Figures 6B,C. Following the assignment of patients based on the median of immune scores, more top 20 mutated genes were observed in those with high immune scores (Figures 6D,E). Figures 6F,G show the comparison of high- and low-risk patients, as well as those with high- and low-immune scores. ATRX, paternally expressed 3 (PEG3), WNK lysine deficient protein kinase 2 (WNK2), neurexin 1 (NRXN1), laminin subunit alpha 2 (LAMA2), and CUB and Sushi multiple domains 2 (CSMD2) demonstrated significant gene mutation differences between high- and low-risk patients (*p* < 0.05) (Figure 6F). Highly mutated genes, such as PEG3, WNK2, NRXN1, and LAMA2 in low-risk patients were also present in patients with high immune scores (Figure 6G). Figure 6H displays that high-risk patients had more TMB (*p* = 0.0025). However, TMB was not associated with OS in STS (Figure 6J). In addition, more TMB was observed in patients with high immune scores; however, the difference was not significant (*p* = 0.23) (Figure 6I).

### Potential Mechanisms Associated With Prognosis

**Figure 7:**
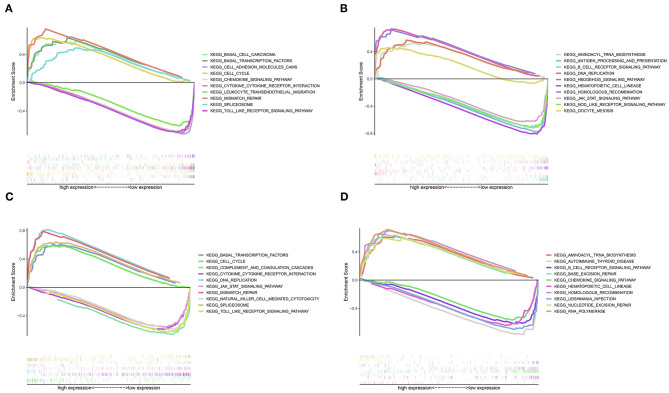
Gene set enrichment analysis for high- and low-risk patients. **(A)** Top 1–5 terms of the GSEA from TCGA dataset. **(B)** Top 6–10 terms of the GSEA from TCGA dataset. **(C)** Top 1–5 terms of the GSEA from the GSE21050 dataset. **(D)** Top 6–10 terms of the GSEA from the GSE21050 dataset.

GSEA revealed that different pathways were altered in high- and low-risk patients, and demonstrated potential mechanisms associated with the biological phenotype (Figure 7). According to the ranking of normalized enrichment scores, KEGG pathways (e.g., cell cycle, mismatch repair, basal transcription factors, spliceosome, aminoacyl tran biosynthesis, and DNA replication) were activated in high-risk patients with shorter OS (Figure 7). However, the activation of immune-related pathways (e.g., the toll-like receptor signaling pathway, Janus kinase/STAT signaling pathway, cytokine-cytokine receptor interaction, B cell receptor signaling pathway, and natural killer cell-mediated cytotoxicity) led to better prognosis in low-risk patients (Figure 7).

### High Relative Methylation Was Observed in Low-Risk Patients

**Figure 8:**
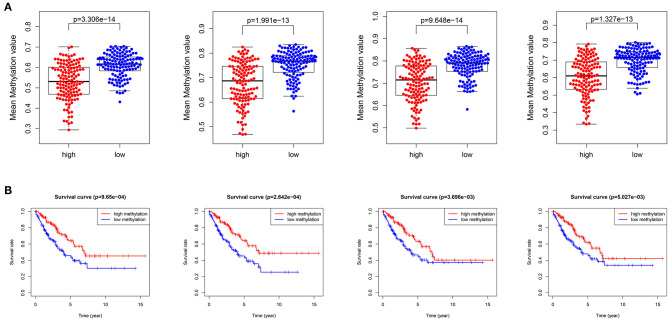
DNA methylation analysis for STS. **(A)** Differences in the mean methylation value in the promoter, body, 3′untranslated regions (3′UTR), and intergenic regions (IGR) (from left to right) between high- and low-risk patients. **(B)** Survival curves for patients with high and low methylation in the promoter, body, 3′UTR, and IGR (from left to right).

After removing the low-quality probes, differential methylation probes were obtained based on the adjusted *p* < 0.05. Subsequently, we extracted the differential probes in the promoter, body, 3′UTR, and IGR. Figure 8A shows that high relative methylation of these four genomic regions was detected in low-risk patients. Notably, high methylation was associated with better OS (Figure 8B). The differential probes were mainly involved in neuroactive ligand-receptor interaction, RAP1 signaling pathway, ECM-receptor interaction, immune-related pathway (e.g., T cell receptor signaling pathway and cytokine-cytokine receptor interaction) and pathways in cancer (e.g., Ras signaling pathway, breast cancer, and basal cell carcinoma) (Table 2).

**Table 2:** The KEGG pathways for the differential probes of methylation.

**Pathway**	**FDR**
Neuroactive ligand-receptor interaction	3.40E-05
Rap1 signaling pathway	7.58E-05
ECM-receptor interaction	0.000226155
Calcium signaling pathway	0.000324435
Olfactory transduction	0.000668637
Bile secretion	0.001056711
Pathways in cancer	0.001266947
Cytokine-cytokine receptor interaction	0.001669184
Adrenergic signaling in cardiomyocytes	0.001669184
Phospholipase D signaling pathway	0.002095849
cAMP signaling pathway	0.003042019
Glutamatergic synapse	0.003538057
PI3K-Akt signaling pathway	0.005381894
Inflammatory mediator regulation of TRP channels	0.006788386
Melanogenesis	0.006788386
Ras signaling pathway	0.008512401
Axon guidance	0.008790691
C-type lectin receptor signaling pathway	0.008790691
Dopaminergic synapse	0.008790691
Estrogen signaling pathway	0.008790691
Vascular smooth muscle contraction	0.009496407
Hypertrophic cardiomyopathy (HCM)	0.009632625
Proteoglycans in cancer	0.00988492
Basal cell carcinoma	0.010425995
Arachidonic acid metabolism	0.011721194
Oxytocin signaling pathway	0.011721194
Dilated cardiomyopathy (DCM)	0.011721194
MAPK signaling pathway	0.01188034
Circadian entrainment	0.01188034
Gastric cancer	0.012601185
Focal adhesion	0.016998021
Renin secretion	0.017058953
Linoleic acid metabolism	0.018485501
Insulin resistance	0.018728758
Human papillomavirus infection	0.018728758
Tight junction	0.019045102
Glycerophospholipid metabolism	0.019618676
Wnt signaling pathway	0.019855177
Hippo signaling pathway	0.019855177
cGMP-PKG signaling pathway	0.022978288
Platelet activation	0.022978288
AGE-RAGE signaling pathway in diabetic complications	0.022978288
Breast cancer	0.025542783
Ether lipid metabolism	0.025994303
Relaxin signaling pathway	0.025994303
Regulation of actin cytoskeleton	0.027185869
Amyotrophic lateral sclerosis (ALS)	0.027202385
Gastric acid secretion	0.028582194
Insulin secretion	0.029401081
Apelin signaling pathway	0.034688902
Phototransduction	0.038659036
Sphingolipid signaling pathway	0.039963306
Pertussis	0.040982726
Parathyroid hormone synthesis, secretion and action	0.042635342
Aldosterone synthesis and secretion	0.045523293
T cell receptor signaling pathway	0.04895089
Fc epsilon RI signaling pathway	0.04895089
Adipocytokine signaling pathway	0.04895089

*KEGG, kyoto encyclopedia of genes and genomes; FDR, false discovery rate*.

### Age, Mitotic Count, and Risk Scores May Be IPF in STS

Figure S5A shows that age (*p* = 0.004), margin status (*p* = 0.009), diagnosis of metastasis (*p* < 0.001), mitotic count (*p* < 0.01), immune scores (*p* = 0.021), and risk scores (*p* < 0.001) were prognostic factors identified in the univariate analysis. The level of immune infiltration measured using the immune scores affected survival in STS. The age, mitotic count and risk scores were considered IPF for STS (Figures S5A,B). Figure S6 shows that risk scores (area under the curve:0.74) had better prognostic ability than immune scores (area under the curve:0.388).

## Discussion

The application of immunotherapy to multiple cancers significantly improved the survival of patients (41). Based on the accumulating evidence and concept of immunotherapy, this approach is effective in the treatment of advanced or metastatic cancers. Immunotherapy has been utilized for the treatment of STS. Previous studies demonstrated that partial patients with STS could benefit from inhibition of PD-1 (42, 43). Immune-related signatures such as tumor inflammation signature (44), immunological constant of rejection (45), and immunophenoscore (46) have been demonstrated as predictors for prognosis and response to immunotherapy in tumors. However, A pan-cancer study revealed that the score of tumor inflammation signature was not associated with prognosis of sarcoma based on univariate Cox regression analysis (44). Therefore, the discovery of new predictive biomarkers of patient response and comprehensive studies of tumor immune microenvironment in STS are crucial for the optimization of immunotherapy in STS (42).

Multi-omic data were utilized to identify potential IRPGs, as well as construct and validate the IGRPM based on WGCNA, univariate, LASSO, and multivariate regression analyses. Finally, five optimal IRPGs and one IGRPM were determined and validated through two datasets involving 573 patients. The IGRPM based on the five IRPGs demonstrated satisfactory performance in predicting the survival rates and risk stratification in patients with STS. IFIH1 (also termed melanoma differentiation-associated gene 5) exhibits an antitumor effect (47) and was a protective gene in STS. CTSG, which possesses the ability to enhance the cytotoxicity of human natural killer cells (48) was overexpressed in low-risk patients with better OS compared with high-risk patients. STC2 and BIRC5 promote metastasis and progression in different types of cancer [e.g., head and neck squamous cell carcinoma (49), hepatocellular carcinoma (50), lung cancer (51), and ovarian tumor (52)] and were overexpressed in high-risk patients. SECTM1, the stimulator of T cells (53), was also identified as a protective gene. Overall, the different prognoses between high- and low-risk patients was consistent with the expression of the five IRPGs, validating the accuracy of the IRGs and IGRPM obtained in this study. To our knowledge, these five IRPGs were firstly combined to construct the IGRPM for STS.

Characterization of tumor immune microenvironment was performed based on two algorithms of ESTIMATE and CIBERSORT. We demonstrated that longer OS in low-risk patients was associated with higher immune activation (including innate and adaptive immunity). Higher immune scores and more activated immune cell infiltrations (e.g., CD8 T cells and activated NK cells) (54, 55) supported this notion and a similar phenomenon was also present in other types of cancer (15, 56). Specifically, low-risk patients had a greater number of innate immune cells owing to the higher expression of TMEM173, triggering innate immunity and innate immunity-related molecules (57). These findings were consistent with those obtained from the algorithm of CIBERSORT. The higher immune activation was also induced by the high expression of immune-stimulating modules [e.g., inducible T cell costimulator [ICOS] (58) and CD80 (59)] and cytokines (e.g., C-C motif chemokine ligand 4 [CCL4], CXCL9, and CXCL10) (60). In addition, the MHC molecules promoted antigen presentation in low-risk patients. Therefore, the lack of immune cells, immune-stimulating molecules, cytokines, and weak antigen-presenting ability led to poor prognosis in high-risk patients.

Immune escape also affects survival (61) and response to immunotherapy in patients with cancer. In this study, tumor cells tended to escape the immune system due to inhibition of immunity in low-risk patients. This effect was demonstrated by high expression of immune inhibiting molecules (e.g., common immune checkpoints and inhibiting cytokines). The expression of immune checkpoints in tumor cells negatively regulates T cells and evades immune killing (62). The high expression of immune checkpoint molecules further suggested that the five identified IRPGs are predictive biomarkers of response to immunotherapy. A recent study reported sex-dependent differences in patient response to immunotherapy in melanoma and non-small-cell lung cancer (41, 63). However, comparison of common checkpoints PD–L1 (CD274), PD1 (PDCD1), CTLA4, and LAG3 between male and female patients reveled that sex is not the main factor for the prediction of response to immune checkpoint inhibitors in STS. This finding was similar to the results reported in a recent study (64). Inhibition of cytokines (e.g., IL10) (45) also inhibited immunity in low-risk patients.

TMB is an emerging biomarker for predicting the effect of immunotherapy in multiple types of cancer (65). The different top 20 gene mutations observed in patients grouped according to risk scores and immune scores led to the hypothesis that low-risk patients may carry more gene mutations. However, this hypothesis was not validated by the value of the TMB. We revealed that high-risk patients (low immune infiltration) had high TMB. This was not consistent with the theory that high mutations (TMB) tend to generate neoantigen epitopes. We hypothesized that high TMB and low antigen-presenting capacity caused low immune infiltration in high risk patients. Of note, both high- and low-risk patients had relative low TMB, which was consistent with a previous study (66). This indicated that the TMB was not suitable for the prediction of response to immunotherapy in STS. Furthermore, the TMB was not associated with survival in STS.

The GSEA revealed that pathways related to cell cycle, DNA replication, and cancer were activated in high-risk patients, leading to poor prognosis. However, the activation of immune-related pathways improved survival. These results also validated the IRPGs and the IGRPM. Furthermore, differences in DNA methylation were investigated between high- and low-risk patients. Relative low methylation in high-risk patients contributed to poor prognosis. The KEGG analysis further revealed the underlying mechanism involved in the effect of DNA methylation on prognosis. In addition, age, mitotic count and risk score were IPF in STS, further validating the prognostic model.

This study had the following clinical implications and strengths. Firstly, although the use of immunotherapy benefited the treatment of cancers, the low response linked to this therapeutic approach limited its use. The identification of “hot tumors” and transformation of “cold tumors” to “hot tumors” could overcome the current predicament (60). Five IRPGs with satisfactory performance in the discrimination of high risk (“cold tumors”) and low risk (“hot tumors”) were identified as potential predictive biomarkers of response to immunotherapy. Low-risk patients not only showed high immune activation but also had inhibition of immunity, especially high expression of checkpoint molecules. Secondly, an increasing number of studies reported that expression of the MHC could predict response to immune checkpoint blockade (67). The significant differences in MHC expression between high- and low-risk patients also suggested the potential use of the five aforementioned IRPGs for the prediction of response to immunotherapy. Thirdly, the differences between high- and low-risk patients revealed by the multi-omic analysis may provide a reference for subsequent studies on the transformation of “cold tumors” to “hot tumors” or improvement of treatment of STS. Fourthly, the combination of different bioinformatics methods increased the reliability of the results.

However, this study was also characterized by some limitations. Firstly, the small sample sizes of the different histological subtypes limited the integrated analysis for each type. Secondly, the stage of STS, which was not available in TCGA dataset, also had a significant impact on prognosis. Subsequent studies focusing on different histological subtypes and the stage of STS are warranted to validate the results of the present study. Thirdly, the ability of five IRPGs to predict prognosis and response to immunotherapy could not be assessed by current methods such as using PD-L1 immunohistochemistry, or Nanostring tumor inflammation signature for lack of data in STS (68). Therefore, five IRPGs also need to be tested in basic experiment and clinical trials.

## Conclusion

In this study, one IGRPM with independent prognostic ability based on five optimal IRPGs (i.e., IFIH1, CTSG, STC2, SECTM1, and BIRC5) were identified and validated in STS. Through the comprehensive study of the tumor immune microenvironment, we demonstrated that these five IRPGs contributed to risk stratification and the identification of patients who are responsive to immunotherapy. Furthermore, the multi-omic analysis revealed the potential mechanisms affecting prognosis, providing additional references for the treatment of STS. More studies focusing on histological subtype were needed to provide more precise treatment.

## Data Availability Statement

The datasets presented in this study can be found in online repositories. The names of the repository/repositories and accession number(s) can be found in the article/Supplementary Material.

## Author Contributions

H-YG, R-XW, and CZ conceived and designed the study. R-XW, H-YG, and L-LL performed the analysis procedures. H-YG, MY, H-CZ, and CZ analyzed the results. H-YG, MY, and CZ contributed analysis tools. H-YG, L-LL, CZ, and R-XW contributed to the writing of the manuscript. H-YG, R-XW, and CZ performed project administration. All authors reviewed the manuscript.

## Conflict of Interest

The authors declare that the research was conducted in the absence of any commercial or financial relationships that could be construed as a potential conflict of interest.
