# Supplementary material for: The Potential of Five Immune-Related Prognostic Genes to Predict Survival and Response to Immune Checkpoint Inhibitors for Soft Tissue Sarcomas Based on Multi-Omic Study
Source: Front Oncol. 2020 Jul 24;10:1317. doi: 10.3389/fonc.2020.01317 (PMC7396489; doi:10.3389/fonc.2020.01317)
Supplement: Supplementary file 1 [file Data_Sheet_1.docx]

**Legends of Supplementary Figure**

**Figure S1: Function enrichment analyses for the genes in blue and brown module.**

**Note:** GO analysis for blue (a) and brown module (c). KEGG pathways for blue (b) and brown module (d).

**Figure S2: Analysis of immune infiltration in the GSE21050 dataset.**

**Note:** Comparison of the immune scores (a) and stromal scores (b) between high- and low-risk patients. (c) Spearman’s correlation of risk scores with the immune scores. (d) Comparison of 22 types of immune cells in high- and low-risk patients. (e) Comparison of the TMEM173, the initiation molecule of innate immunity between high- and low-risk patients. Comparison of the other molecules of innate immunity (f), major histocompatibility complex (MHC) molecules (g), and immune-stimulating molecules (h), and cytokines (i) between high- and low-risk patients. Low-risk patients exhibited high expression of most of these molecules. Relationships of immune infiltration (immune scores) and immune-stimulating molecules (j). Immune infiltration was positively correlated with most immune-stimulating molecules. ImmS represents the immune scores. ***, p<0·001; **, 0·001<p<0·01; *, 0·01<p<0·05; ns (not significant), p>0·05.

**Figure S3: Inhibition of immunity in low-risk patients from GSE21050 dataset.**

**Note:** (a): Differences in common immune checkpoint molecules between high- and low-risk patients. (b) Differences in immune-inhibiting molecules between high- and low-risk patients. (c) Relationships of immune infiltration (immune scores) and immune-inhibiting molecules. Immune infiltration was positively correlated with most immune-inhibiting molecules. ImmS represents the immune scores. ***, p<0·001; **, 0·001<p<0·01; *, 0·01<p<0·05; ns (not significant), p>0·05.

**Figure S4: Sex-dependent differences in common immune checkpoint molecules.**

**Figure S5: Forest plot of the univariate and multivariate analyses for the clinical factors in STS.**

**Note:** (a): Forest plot of the univariate analyses. (b): Forest plot of the multivariate analyses.

**Figure S6: The receiver operating characteristic (ROC) curves of risk score and immune score in TCGA dataset.**

**
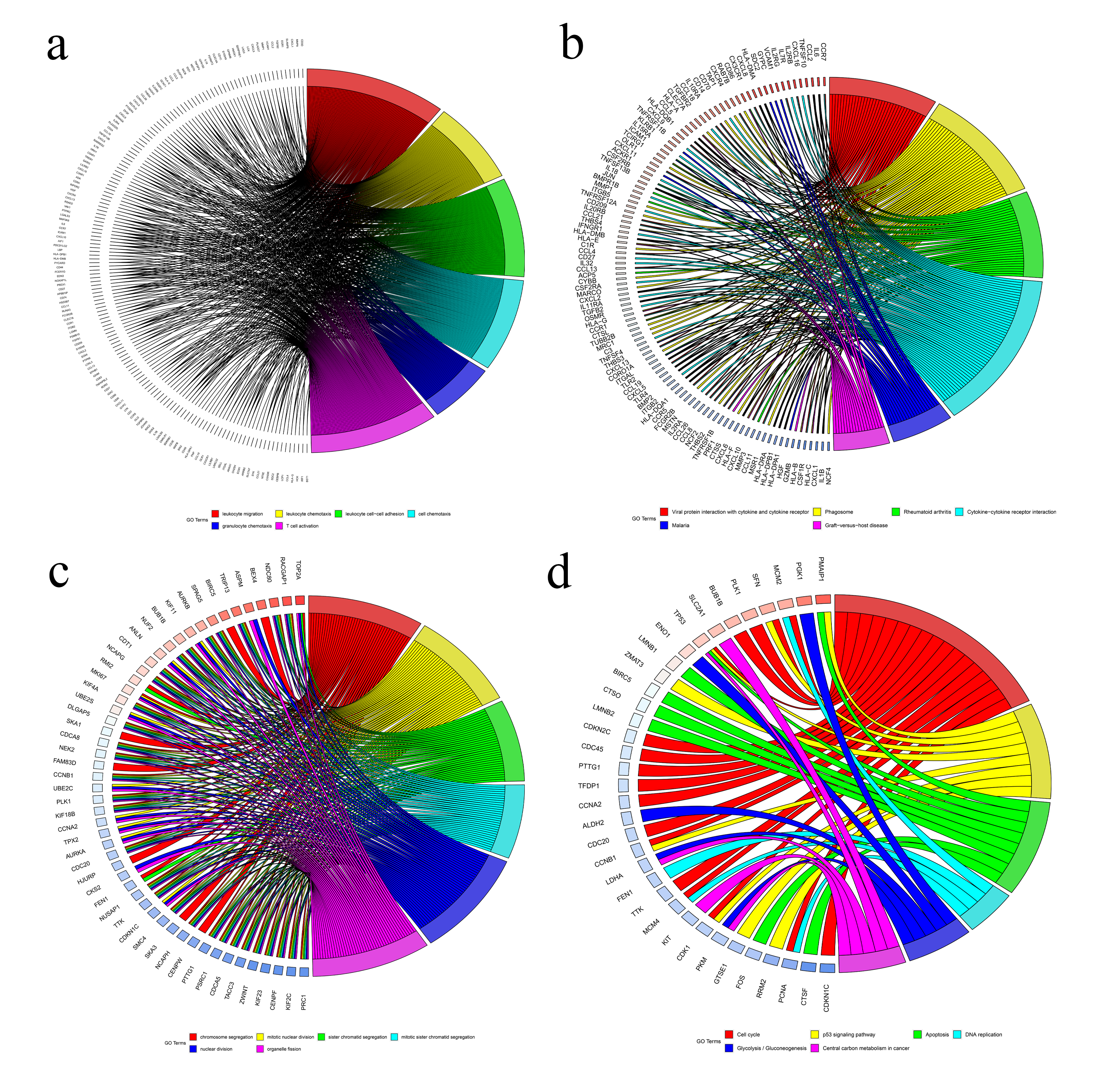
**

**Figure S1: Function enrichment analyses for the genes in blue and brown module.**

**Note:** GO analysis for blue (a) and brown module (c). KEGG pathways for blue (b) and brown module (d).

**
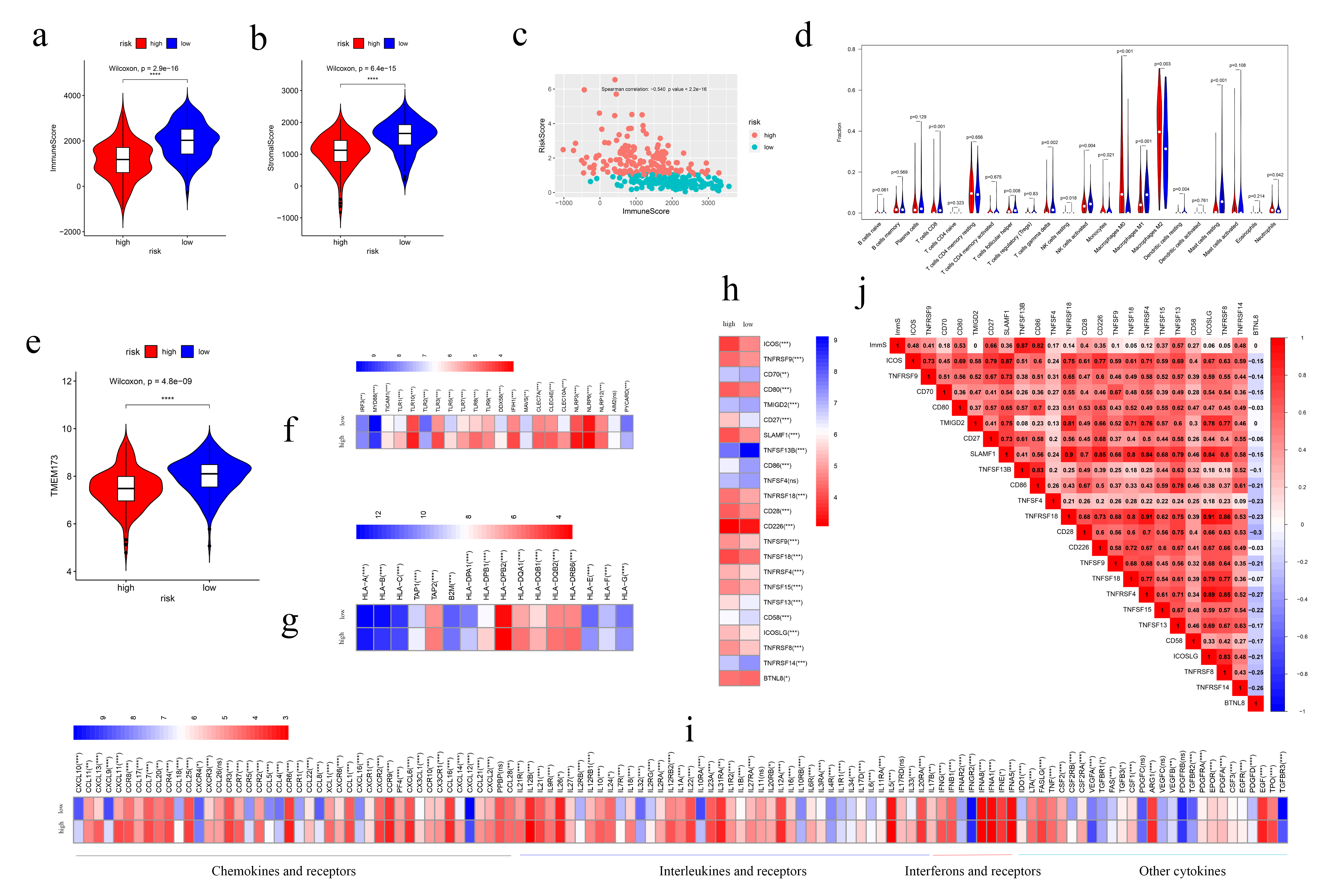
**

**Figure S2: Analysis of immune infiltration in the GSE21050 dataset.**

**Note:** Comparison of the immune scores (a) and stromal scores (b) between high- and low-risk patients. (c) Spearman’s correlation of risk scores with the immune scores. (d) Comparison of 22 types of immune cells in high- and low-risk patients. (e) Comparison of the TMEM173, the initiation molecule of innate immunity between high- and low-risk patients. Comparison of the other molecules of innate immunity (f), major histocompatibility complex (MHC) molecules (g), and immune-stimulating molecules (h), and cytokines (i) between high- and low-risk patients. Low-risk patients exhibited high expression of most of these molecules. Relationships of immune infiltration (immune scores) and immune-stimulating molecules (j). Immune infiltration was positively correlated with most immune-stimulating molecules. ImmS represents the immune scores. ***, p<0·001; **, 0·001<p<0·01; *, 0·01<p<0·05; ns (not significant), p>0·05.


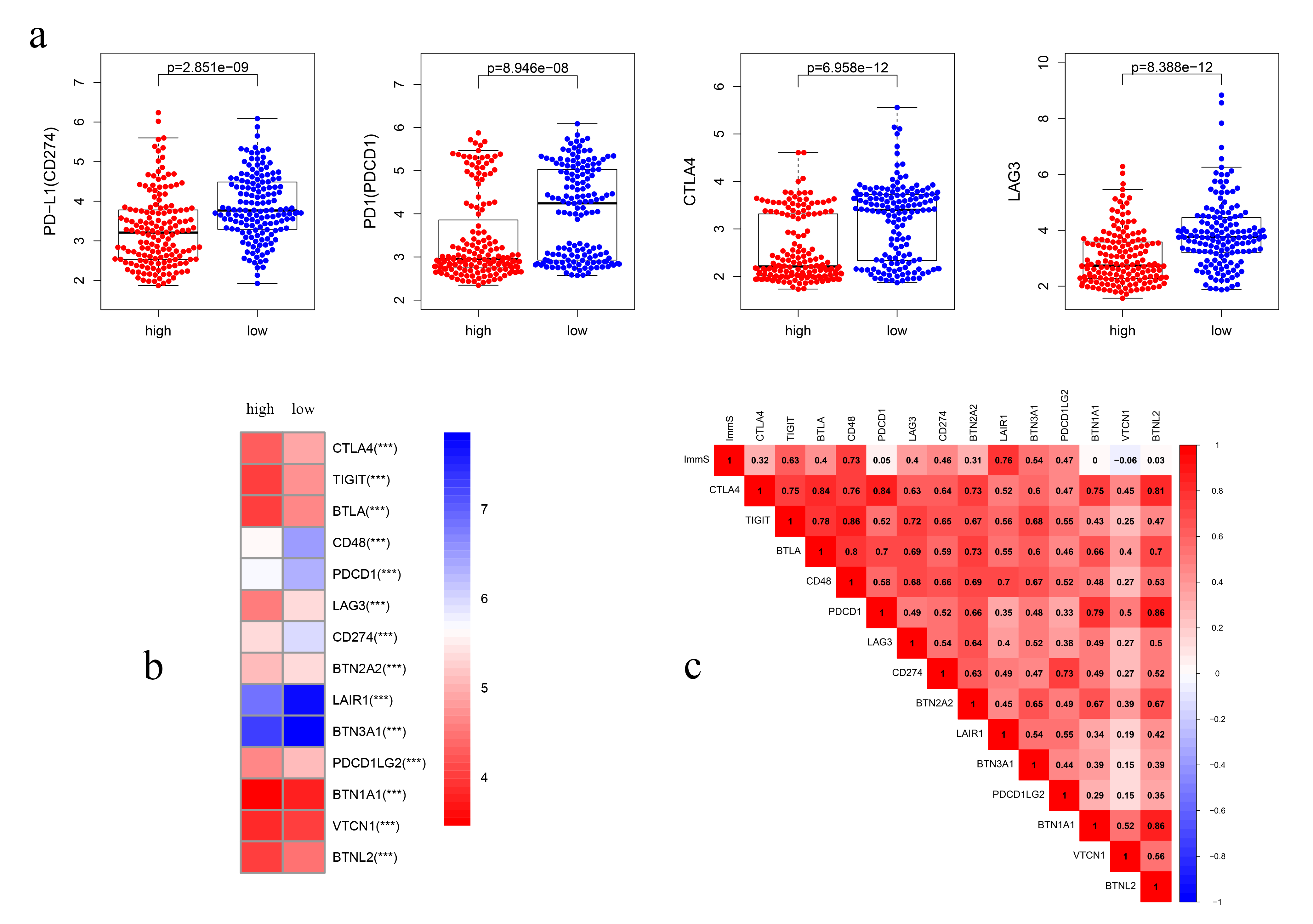


**Figure S3: Inhibition of immunity in low-risk patients from GSE21050 dataset.**

**Note:** (a): Differences in common immune checkpoint molecules between high- and low-risk patients. (b) Differences in immune-inhibiting molecules between high- and low-risk patients. (c) Relationships of immune infiltration (immune scores) and immune-inhibiting molecules. Immune infiltration was positively correlated with most immune-inhibiting molecules. ImmS represents the immune scores. ***, p<0·001; **, 0·001<p<0·01; *, 0·01<p<0·05; ns (not significant), p>0·05.


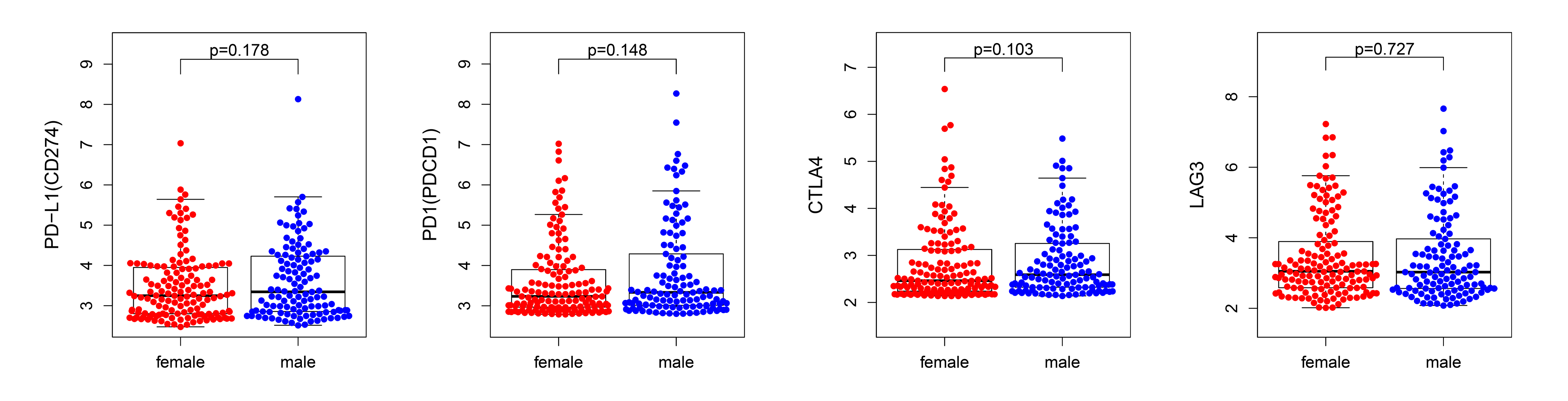
**Figure S4: Sex-dependent differences in common immune checkpoint molecules.**


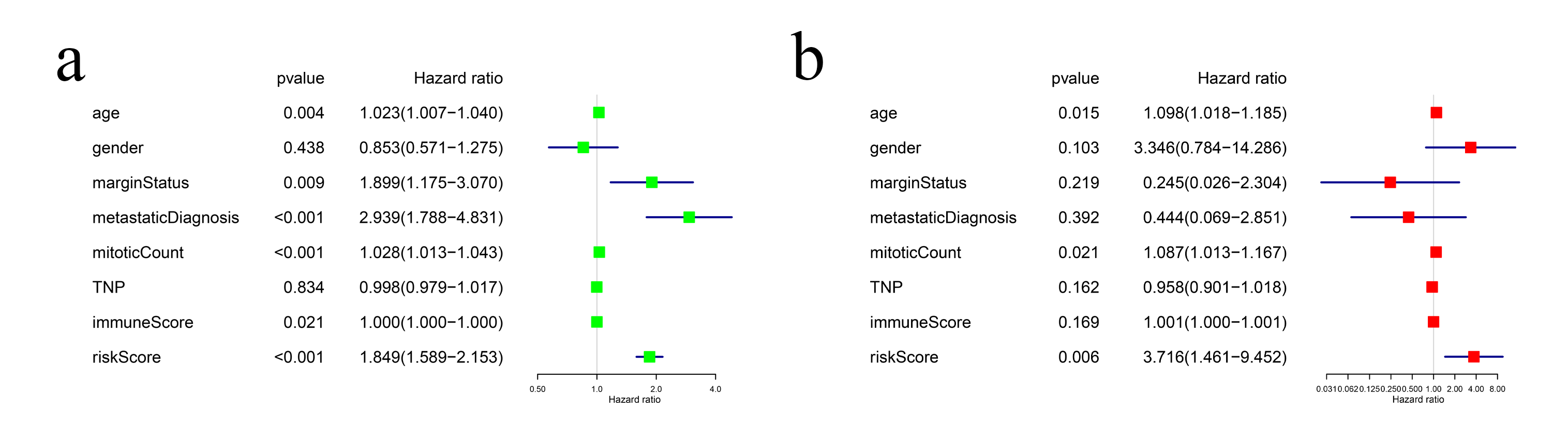


**Figure S5: Forest plot of the univariate and multivariate analyses for the clinical factors in STS.**

**Note: (a): Forest plot of the univariate analyses. (b): Forest plot of the multivariate analyses.**


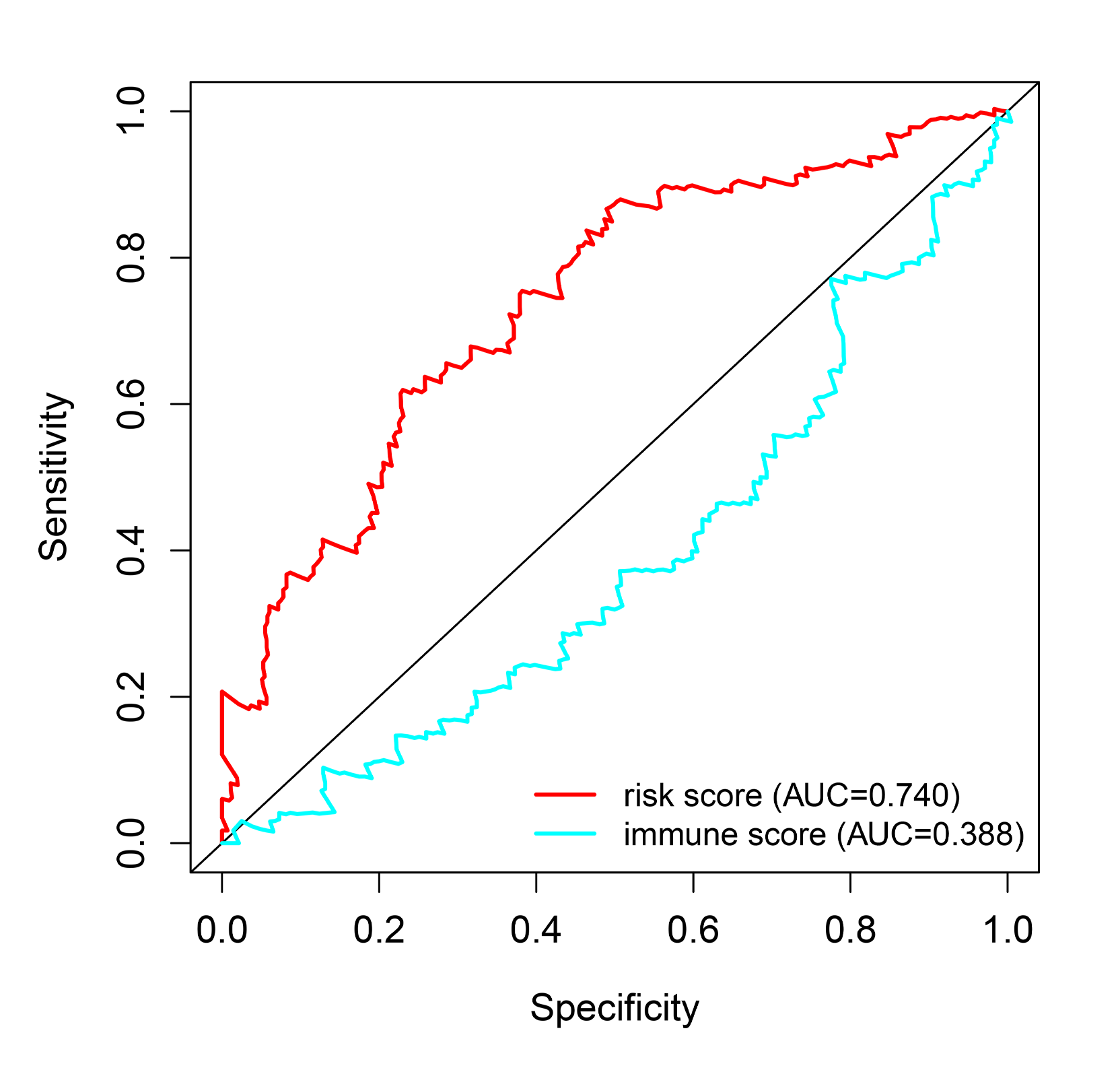


**Figure S6: The receiver operating characteristic (ROC) curves of risk score and immune score in TCGA dataset.**
